# Skeletal callus formation is a nerve‐independent regenerative response to limb amputation in mice and *Xenopus*

**DOI:** 10.1002/reg2.39

**Published:** 2015-08-26

**Authors:** Shinichirou Miura, Yumiko Takahashi, Akira Satoh, Tetsuya Endo

**Affiliations:** ^1^ Division of Liberal Arts and SciencesAichi Gakuin UniversityNissinAichi470‐0195Japan; ^2^ Research Core for Interdisciplinary SciencesOkayama UniversityOkayama700‐8530Japan

**Keywords:** Fracture healing, limb regeneration, macrophage, nerve, wound healing

## Abstract

To clarify the mechanism of limb regeneration that differs between mammals (non‐regenerative) and amphibians (regenerative), responses to limb amputation and the accessory limb inducible surgery (accessory limb model, ALM) were compared between mice and *Xenopus*, focusing on the events leading to blastema formation. In both animals, cartilaginous calluses were formed around the cut edge of bones after limb amputation. They not only are morphologically similar but show other similarities, such as growth driven by undifferentiated cell proliferation and macrophage‐dependent and nerve‐independent induction. It appears that amputation callus formation is a common nerve‐independent regenerative response in mice and *Xenopus*. In contrast, the ALM revealed that the wound epithelium (WE) in *Xenopus* was innervated by many regenerating axons when a severed nerve ending was placed underneath it, whereas only a few axons were found within the WE in mice. Since nerves are involved in induction of the regeneration‐permissive WE in amphibians, whether or not nerves can interact with the WE might be one of the key processes separating successful nerve‐dependent blastema formation in *Xenopus* and failure in mice.

## Introduction

Limb regeneration in amphibians is a typical example of epimorphosis, which is associated with formation of a regeneration blastema (see Carlson 2007). Among amphibians, successful limb regeneration appears only in urodeles. Numerous classical and molecular biology studies have revealed that limb regeneration consists of a series of critical steps, wound healing, blastema formation and pattern formation, and that many factors are found to be involved in each step (reviewed by Mescher 1996; Bryant et al. 2002; Gardiner et al. 2002). Our group has reexamined and demonstrated that these steps transition in a stepwise manner, being mediated by defined factors (the stepwise model; Endo et al. 2004). Factors from nerves affect mesenchymal cells around the wounded site and induce blastema formation. Positional diversity among mesenchymal cells is necessary for the blastema to develop a patterned limb.

The importance of the nerve in blastema formation is conserved in some anuran amphibians too. Instead of regenerating a perfectly patterned limb, an African clawed frog (*Xenopus laevis*) forms a cartilaginous rod surrounded by connective tissues and skin, called a spike, in response to limb amputation (Dent 1962). Spike formation is thought to be an example of partial regeneration, since it is accompanied by blastema formation but lacks expression of some patterning genes, such as *shh* (Endo et al. 2000). When a limb is denervated at the time of amputation, a blastema fails to form, and the wound of the amputation surface just heals, suggesting that nerves play an essential role for induction of a spike‐forming blastema as well as in urodeles (Korneluk et al. 1982; Endo et al. 2000).

In addition to nerves, recent studies suggest the importance of inflammatory reactions for regenerative responses in several systems (King et al. 2012; Kyritsis et al. 2012; Godwin et al. 2013; Love et al. 2013; Mescher et al. 2013; Petrie et al. 2014). Among inflammation‐related cells, macrophages are a signaling source for promotion and resolution of inflammation (reviewed by Koh & DiPietro 2011) and are involved in the initiation of regenerative responses. In axolotls, systemic depletion of macrophages at an early phase of limb regeneration results in failure of blastema formation (Godwin et al. 2013). The presence of macrophages is also important in tissue repair, such as muscle injury (Ruffell et al. 2009) and bone fracture (Raggatt et al. 2014).

Compared to amphibians, the regenerative ability in a mammalian limb is largely restricted. The only level at which mammals can regenerate limb structures is the digit tip level. In mice, the most distal phalanx is regenerative at the distal level but non‐regenerative at the proximal level of this bone (Borgens 1982; Han et al. 2008; Takeo et al. 2013) as well as at more proximal levels, such as long bones of the zeugopod and stylopod. Limb amputation at non‐regenerative levels along the proximal−distal limb axis results in wound closure over the amputation surface. Therefore the blastema is never formed on the stump unlike in amphibians. In addition to skin wound healing there is tissue repair in the stump. A typical response to amputation in rodents is formation of a bony callus at the cut edge of the amputated bone, which is especially evident in neonates (Libbin & Weinstein 1986; Masaki & Ide 2007; Ide 2012). It was reported that the growth plate is occasionally regenerated in rats (Libbin & Weinstein 1986), although the length of the bone does not seem to recover to the original length.

The purpose of our research was to explore regenerative failure in mice with the goal of enhancing mammalian regenerative responses. To approach this goal, we compared responses to limb amputation in mice with those in *Xenopus* froglets, focusing on the events leading to blastema formation. Although amputated *Xenopus* limbs show only partial regenerative responses, the events leading to blastema formation seem to be normal (Suzuki et al. 2006). In this study, we report that formation of the amputation callus around the cut edge of the bones is a common response to limb amputation in mice and *Xenopus*. In both animals, it occurs macrophage‐dependently but nerve‐independently. It is followed by blastema formation in *Xenopus* but not in mice. To explain this difference, we hypothesize that one of the key processes to separate successful nerve‐dependent blastema formation in *Xenopus* and failure in mice is whether or not regenerating nerves can interact with the wound epithelium (WE). In *Xenopus*, regenerating axons innervate into the WE and convert it to the regeneration‐permissive WE, the apical epithelial cap (AEC). In contrast, the WE formed in amputated mouse limbs was innervated by only a few nerve fibers, suggesting that the nerve‐dependent conversion to the AEC did not occur. As has been hypothesized (Tassava & Olsen 1982), this might be one of the reasons that mice cannot form a blastema and ultimately fail to regenerate a limb.

## Results

### Limb amputation induces formation of the amputation callus in mice

**Figure 1:**
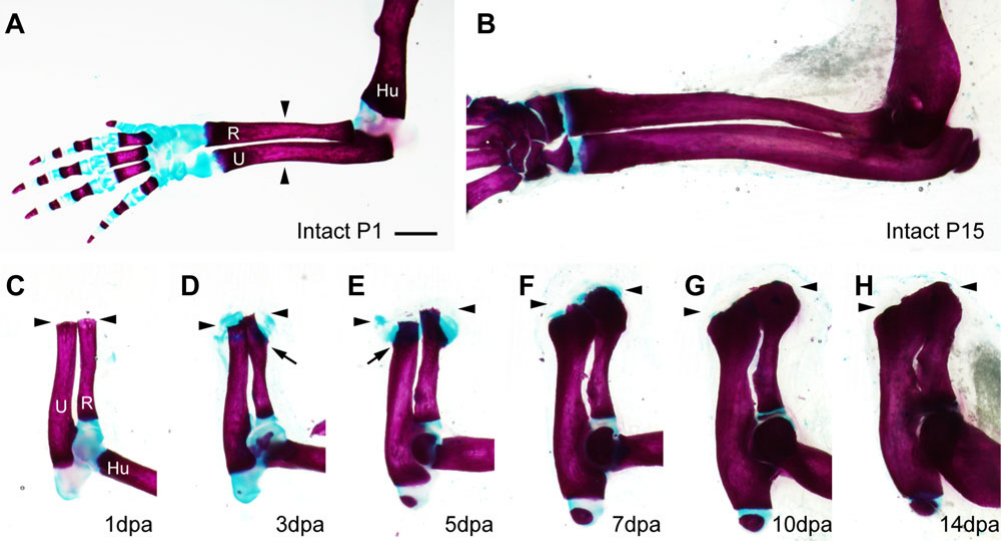
Time course of amputation callus formation in P1 neonatal mice. The whole‐mount samples were stained with alcian blue and alizarin red. (A), (B) Intact forelimbs of P1 (A) and P15 (B) neonates. (C)−(H) Formation of the amputation callus at 1 dpa (C), 3 dpa (D), 5 dpa (E), 7 dpa (F), 10 dpa (G), and 14 dpa (H). The arrowheads indicate the amputation level. The arrows indicate the points where ossification begins in the cartilaginous calluses. Skeletal tissues are indicated; R, radius; U, ulna; Hu, humerus. Scale bar 1 mm (applicable to all photographs).

A forelimb was amputated at the middle of the forearm level at postnatal day 1 (P1) to observe temporal changes in skeletal morphology. Bones in a limb at P1 are still immature for the most part (Fig. 1A), compared to a more mature limb at P15 (Fig. 1B). Although the diaphyses of long bones and phalanges are already calcified, the whole epiphyses are still cartilaginous.

**Figure 2:**
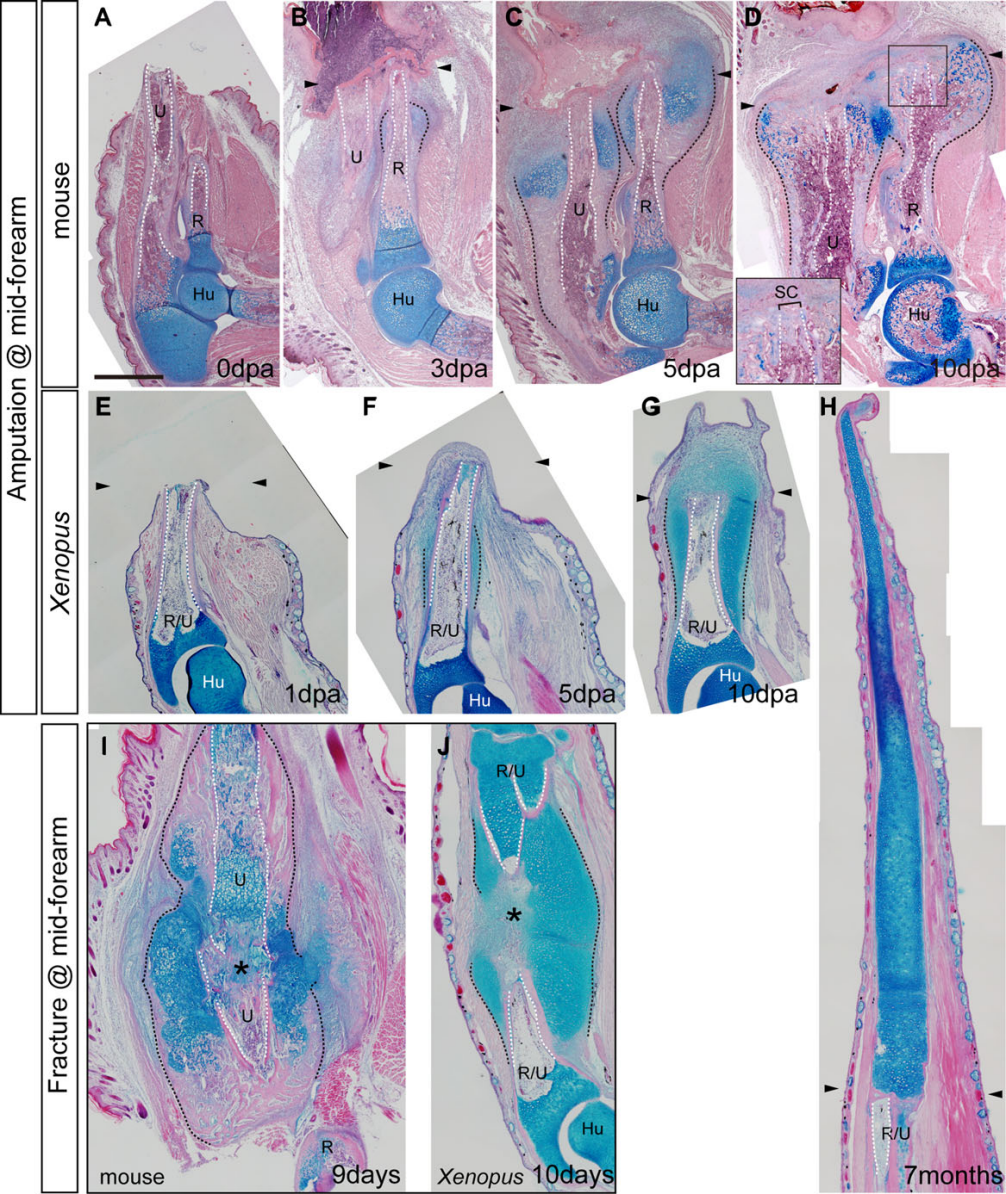
Comparison of regenerative responses in mice and Xenopus. Tissue sections were stained with hematoxylin and eosin and alcian blue. (A)−(D) Amputated forelimbs of P1 mice at 0 dpa (A), 3 dpa (B), 5 dpa (C), and 10 dpa (D). (E)−(H) Amputated forelimbs of Xenopus froglets at 1 dpa (E), 3 dpa (F), 5 dpa (G), and after 7 months (H). The inset of (D) is a highly magnified image of the squared area and shows the skeletal cap that seals the medullary cavity of the amputated bones. (I), (J) Fracture healing in P1 mice (9 days) (I) and Xenopus froglets (10 days) (J). The arrowheads indicate the amputation level. The asterisks indicate the gap between broken bones. Skeletal tissues are indicated; R, radius; U, ulna; R/U, radio‐ulna; Hu, humerus; SC, skeletal cap. The amputation calluses and the outline of the medullary cavity are delineated by black and white dotted lines, respectively. Scale bar 500 μm (applicable to all photographs).

A typical response to limb amputation in P1 neonatal mice is cartilaginous callus formation which subsequently undergoes ossification. The cartilaginous amputation calluses were observed around the cut edge of the radius and ulna at 3 days post‐amputation (dpa) (Fig. 1D; compare with Fig. 1C at 1 dpa). A small protuberance of the calcified area was seen in the proximal region of the amputation callus in the radius (indicated by an arrow in Fig. 1D). At 5 dpa, the amputation calluses eventually grew into a bulbous shape (Fig. 1E). The calcified area at the proximal callus appeared in the ulna too (indicated by an arrow in Fig. 1E). By 7 dpa, almost all of the amputation calluses were replaced by ossified bone, although small areas of alcian blue staining remained at the distal tip (Fig. 1F). By 10 dpa, the alcian blue stain had disappeared around the amputation callus (Fig. 1G) and the proximal part of the amputated bones was thickened. At 14 dpa, the size of the amputation callus did not appear to have changed, but it was less prominent because the proximal bone had become thicker (Fig. 1H). Amputation calluses, however, were still slightly thicker than control intact bones (compare with Fig. 1B). It should be noted that the amputated bones showed little distal elongation. Although the cut end of the radius gradually became more distal to that of the ulna (Fig. 1C−H), this elongation depended on the growth plate of the proximal epiphysis, but not on the growth of the amputation callus, as is evident in histological sections (see below, Fig. 2).

### Comparison of the regenerative responses in mice and *Xenopus*

To characterize the similarities and differences between regenerative and non‐regenerative animals, we analyzed histological tissue sections to compare the response of amputated limbs in P1 mice to that in *Xenopus* froglets.

Histological sections clearly show that the amputation callus in mice enlarged laterally, whereas there was no evidence of any bony structures being formed distal to the amputation plane. At 3 dpa in the mouse, alcian blue stained cartilaginous calluses started to form from the periosteum near the amputation plane (Fig. 2B; compare to Fig. 2A at 0 dpa). Cartilaginous regions expand laterally and develop into bulbous‐shaped bony calluses (Fig. 2B−D). Although faint alcian blue staining was sometimes observed in the tissues distal to the amputation plane, we did not observe formation of additional structure other than the skeletal cap that was formed at the cut edge of the bone by 10 dpa, which sealed the medullary cavity (Fig. 2D).

In contrast to the response in the mouse, amputation of a *Xenopus* limb resulted in a robust chondrogenic response, leading to formation of a hypomorphic spike distal to the amputation plane (Fig. 2E−H). As observed in mice, cartilaginous callus formation from the periosteum was one of earliest events in a *Xenopus* amputated limb. An alcian‐blue‐positive region appeared from the middle of the remaining part of the amputated bones and spread widely along the bone by 5 dpa (Fig. 2F; compare to Fig. 2E at 1 dpa). This growth developed into a cartilaginous structure that not only expanded laterally but also elongated distally from the amputation plane (Fig. 2G). By this time, appearance of a regeneration blastema became noticeable. The blastema kept elongating, and a distally elongated cartilage eventually formed a cartilaginous spike (Fig. 2H). Although a very thin layer of cortical bone was seen on the surface of the proximal part of the spike, there was no other indication of ossification in the distal part. On the other hand, a laterally expanded cartilage around the stump bones was progressively ossified.

We also compared the morphology between the amputation callus and fracture callus. When an incision was made in the middle of the radius and ulna in mice and radio‐ulna in *Xenopus*, cartilaginous calluses were formed from both cut ends of the skeletal defect, bridging the broken bones (Fig. 2I, J). The morphology of the fracture calluses on the proximal broken bones was similar to the amputation callus except the fracture callus was slightly bigger than the amputation callus.

### Comparison of the pattern of proliferation during amputation callus and spike formation

**Figure 3:**
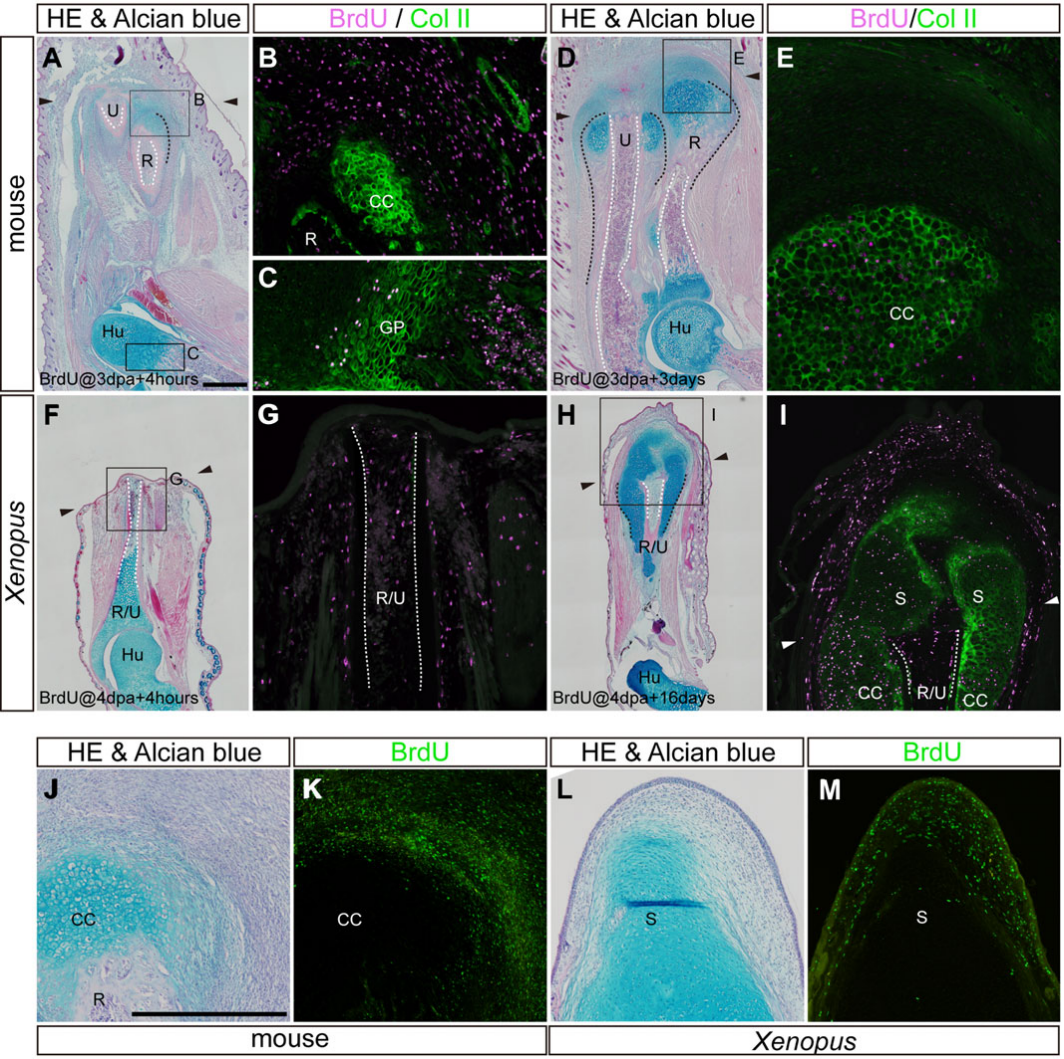
Lineage tracing of BrdU‐labeled proliferating cells in amputated limbs. (A)−(E) In mice, BrdU was injected intraperitoneally at 3 dpa, and tissue samples were fixed 4 h (A)−(C) and 3 days (D), (E) after injection. (F)−(I) In Xenopus, BrdU was injected intraperitoneally at 4 dpa, and tissue samples were fixed 4 h (F), (G), and 16 days (H), (I), after injection. In both animals, adjacent sections were stained by hematoxylin and eosin and alcian blue (A, D, F, H) and anti‐BrdU antibody (magenta in B, C, E, G, I). BrdU‐stained sections were also stained by anti‐type II collagen antibody (green in B, C, E, G, I). Areas in (B), (C), (E), (G), and (I) are high magnifications of the squared areas in (A), (D), (F) and (H). (J)−(M) Repeated BrdU injection was performed in 5‐dpa mice (J), (K) and 3 weeks post‐amputation Xenopus (L), (M). Adjacent sections were stained by hematoxylin and eosin and alcian blue (J, K) and anti‐BrdU antibody (green in K and M). The arrowheads indicate the amputation level. Skeletal tissues are indicated; R, radius; U, ulna; R/U, radio‐ulna; Hu, humerus; GP, growth plate; CC, cartilaginous callus; S, spike cartilage. The amputation calluses and the outline of the medullary cavity are delineated by black and white dotted lines, respectively. Scale bars in (A) (applicable to D, F, H) and (J) (applicable to K−M) 500 μm.

Amputation‐induced cartilaginous calluses in mice enlarged laterally by addition of proliferating mesenchymal cells surrounding the callus rather than proliferation of chondrocytes within the callus. 5‐Bromo‐2'‐deoxyuridine (BrdU) was injected intraperitoneally in neonatal mice at 3 dpa when the cartilaginous callus was rapidly growing, and limbs were fixed either 4 h (Fig. 3A−C) or 3 days (Fig. 3D, E) later. After 4 h of labeling, few cells in the type II collagen‐positive amputation callus incorporated BrdU; instead, bone marrow cells (not shown) and mesenchymal cells around the callus incorporated BrdU (Fig. 3B), indicating that chondrocytes in the amputation callus were not proliferative. When we examined the distribution of BrdU‐positive cells 3 days after injection (at 6 dpa), we observed BrdU‐positive cells within the type II collagen‐positive amputation callus (Fig. 3D, E), suggesting that proliferating mesenchymal cells that had been labeled at the time of BrdU injection (3 days earlier) had been incorporated into the callus and had differentiated into chondrocytes. On the other hand, in the epiphysis of uninjured bones, BrdU was incorporated into type II collagen‐positive chondrocytes of the growth plate (Fig. 3A, C), indicating that there is a difference between amputation callus growth and uninjured bone elongation.

A cartilaginous spike in *Xenopus* elongated distally by adding proliferating mesenchymal cells to the distal end rather than by proliferation of chondrocytes at a more proximal level of the spike, in a similar fashion to the growth of the amputation callus in the mouse. First, BrdU was injected intraperitoneally in *Xenopus* froglets at 4 dpa when alcian‐blue‐positive regions were just appearing around the amputated bone, and limbs were fixed in 4 h (Fig. 3F, G) or after 16 days (Fig. 3H, I). Four hours after BrdU injection, type II collagen was localized only in the skeletal cartilages such as the articular cartilage (not shown) and largely overlapped with alcian‐blue‐positive regions (see Fig. 3F). Therefore BrdU‐positive cells around the cut edge at this point were type II collagen‐negative, indicating that there were no proliferating chondrocytes near the amputated bone (Fig. 3G). Instead, BrdU was incorporated into bone marrow cells and mesenchymal cells in degenerating muscle tissues, periosteum, and connective tissues. Sixteen days after injection (20 dpa), BrdU‐positive cells were found in the distal mesenchyme and spike cartilages of the blastema and the cartilaginous calluses of the stump (Fig. 3H, I). These results suggest that proliferating mesenchymal cells at 4 dpa eventually differentiated into chondrocytes in the spike and cartilaginous calluses. Second, BrdU was injected every 4 h for 12 h (three times) into 5 dpa mice and 3 weeks post‐amputation *Xenopus* to visualize the regions of proliferating and non‐proliferating cells more clearly. In both animals, BrdU was detected mainly in the mesenchyme surrounding the newly formed cartilage (Fig. 3J−M), supporting the previous data.

### Formation of an amputation callus and fracture callus are inhibited in clodronate‐liposome injected limbs in both mice and *Xenopus*

Recently macrophages have been suggested to be involved in appendage regeneration. In axolotl limbs, the depletion of macrophages by intravenous injection of liposome‐encapsulated clodronate (Clo‐lipo) resulted in failure of blastema formation (Godwin et al. 2013). Thus we tested if macrophage depletion affects amputation callus formation in mice and *Xenopus*.

**Figure 4:**
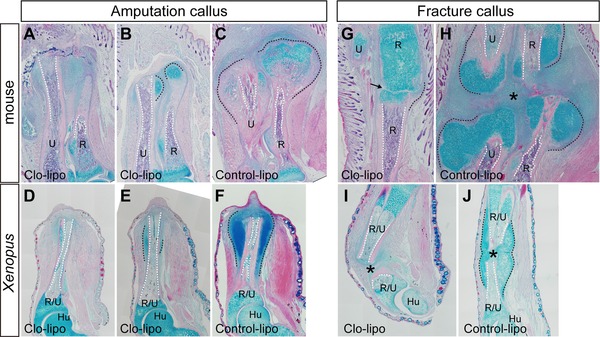
Formation of the amputation and fracture calluses in Clo‐lipo injected mice and Xenopus. Tissue sections were stained with hematoxylin and eosin and alcian blue. (A)−(F) Clo‐lipo injection results in inhibition of amputation calluses (A, B, D, E) in mice (7 dpa) (A−C) and Xenopus (10 dpa) (D−F), compared to controls (C, F). Two typical phenotypes are shown. In (A) and (D), no round‐shaped chondrocytes are found around the cut edge of the amputated bones. In (B) and (E), the size of the cartilaginous calluses is dramatically reduced. In Xenopus, the thickened epidermis is formed on the amputation plane in both strong inhibition phenotype (D) and reduced‐size phenotype (E), although a blastema is not formed. In a control limb (F), a small blastema already appears. (G)−(J) Clo‐lipo injection also inhibits formation of the fracture callus in mice (7 days) (G, H) and Xenopus (14 days) (I, J). (H), (J) Control‐lipo injected limbs. The granulation tissue between the broken bones is also dramatically reduced by Clo‐lipo injection (G, I). (A), (C), (G)−(J) Locally injected samples. (D)−(F) Intraperitoneally injected samples. Skeletal tissues are indicated; R, radius; U, ulna; R/U, radio‐ulna; Hu, humerus. The amputation calluses and the outline of the medullary cavity are delineated by black and white dotted lines, respectively. An arrow in (G) and an asterisk in (H) indicate the gap between the broken bones. Scale bar 500 μm.

In mouse neonates, Clo‐lipo injection drastically inhibited amputation callus formation (Table 1). Instead of intravenous injection, we performed direct injection of Clo‐lipo into the amputated limb stump prior to limb amputation. In Clo‐lipo injected limbs, either the typical round‐shaped chondrocytes were not found around the amputated bone (Fig. 4A) or the size of the cartilaginous callus was severely reduced (Fig. 4B), while a part of the callus was already ossified in the control limbs (*P *< 0.01) (Fig. 4C). We confirmed that expression of macrophage markers (CD68 and F4/80) was decreased in the Clo‐lipo injected mouse limbs by quantitative reverse transcription polymerase chain reaction (not shown), indicating that inhibition of the amputation callus formation was accompanied by reduction of macrophages. Therefore it is suggested that macrophages are involved in the initiation of the amputation callus formation. We also tested intraperitoneal injection of Clo‐lipo, but did not observe any effects on callus formation (not shown).

**Table 1:** Formation of the amputation callus in clodronate‐liposome (Clo‐lipo) injected animals

				Amputation callus		
	Day after surgery	Number of samples	Spike^a^	Normal	Partially formed^b^	Not formed^c^
Mouse						
Local injection						
Clo‐lipo	7 days	11	−	1 (9%)	10 (91%)	0 (0%)
Control‐lipo	7 days	5	−	5 (100%)	0 (0%)	0 (0%)
*Xenopus*						
Local injection						
Clo‐lipo	14 days	5	−	0 (0%)	3 (60%)	2 (40%)
Control‐lipo	10 days	7	−	7 (100%)	0 (0%)	0 (0%)
Intraperitoneal injection						
Clo‐lipo	10 days	4	−	0 (0%)	3 (75%)	1 (25%)
	20–60 days	5	0	0 (0%)	2 (40%)	3 (60%)
Control‐lipo	10 days	9	−	9 (100%)	0 (0%)	0 (0%)
	20–30 days	3	3	3 (100%)	0 (0%)	0 (0%)

^a^Spike formation was counted in animals that were raised for a long time after amputation, so that a spike is easily recognizable in control animals.

^b^Small cartilaginous calluses are formed, but the size of the calluses is dramatically reduced.

^c^No round‐shaped chondrocytes are found ectopically.

In *Xenopus* spike formation, Clo‐lipo treatment prior to limb amputation inhibited blastema formation as was reported for axolotl limb regeneration (Table 1). Although a thickened epidermis formed at the center of the amputation surface, blastema outgrowth did not occur (Fig. 4D, E; Table 1). Importantly, amputation callus formation was also inhibited in all cases (Table 1; Fig. 4D, E; compare with Fig. 4F as control [*P* < 0.01]). Intraperitoneal injection of Clo‐lipo resulted in the same outcome as local injection in *Xenopus* amputation callus. The photographs in Figure 4D−F are all from intraperitoneally injected animals (the results from day 10 samples are significant at the 0.01 level).

Clo‐lipo also has an inhibitory effect on fracture callus formation when injected into the limb locally (*P* < 0.001 in both mice and *Xenopus* experiments) (Fig. 4G−J and Table 2). In fracture healing of forearm bones, Clo‐lipo inhibited formation of the hematoma and cartilaginous calluses (fracture calluses) in/around the gap between the broken bones in both mice (Fig. 4G, H) and *Xenopus* (Fig. 4I, J). Intraperitoneal injection of Clo‐lipo had no effect on fracture callus formation.

**Table 2:** Formation of the fracture callus in clodronate‐liposome (Clo‐lipo) injected animals

	Day after surgery	Number of samples	Normal	Partially formed^a^	Not formed^b^
Mouse					
Local injection					
Clo‐lipo	7 days	6	0 (0%)	4 (67%)	2 (33%)
Control‐lipo	7 days	8	8 (100%)	0 (0%)	0 (0%)
*Xenopus*					
Local injection					
Clo‐lipo	14 days	6	0 (0%)	1 (17%)	5 (83%)
Control‐lipo	14 days	8	8 (100%)	0 (0%)	0 (0%)
Intraperitoneal injection					
Clo‐lipo	10 days	5	5 (100%)	0 (0%)	0 (0%)
Control‐lipo	10 days	5	5 (100%)	0 (0%)	0 (0%)

^a^Small cartilaginous calluses are formed, but the size of the calluses is dramatically reduced. Chondrocytes are found ectopically.

^b^No round‐shaped chondrocytes are found ectopically.

### Initiation of amputation callus formation appears to be nerve‐independent

It has been known that there is a relationship between nerves and regenerative responses in many tissues and organs (see Carlson 2007). To investigate if nerves affect amputation callus formation, we performed denervation of amputated limbs in mice and *Xenopus*.

**Figure 5:**
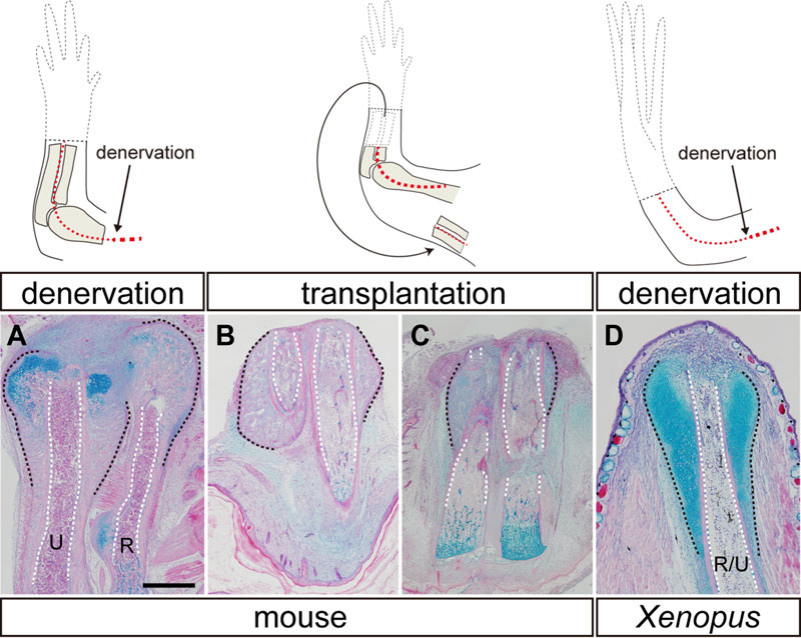
Amputation callus formation in denervated limbs of mice and Xenopus. Illustrations in the upper row show the surgeries that each limb was given. Tissue sections were stained with hematoxylin and eosin and alcian blue. Amputation calluses were formed in both mice at day 7 (A)−(C) and Xenopus at day 10 (D) even after limb denervation (A, D) or limb tissue transplantation (B, C). Significant differences were found between normal (see Fig. 2) and denervated limbs (A, D). Calluses were also formed in the transplanted limb tissues but the size was various (B, normal; C, reduced). Radius and ulna were indistinguishable in the transplants. The amputation calluses and the outline of the medullary cavity are delineated by black and white dotted lines, respectively. Skeletal tissues are indicated; R, radius; U, ulna; R/U, radio‐ulna. Scale bar 500 μm (applicable to all photographs).

Amputation callus formation occurred in both mouse and *Xenopus* limbs that had been surgically denervated (Fig. 5 and Table 3). In mice, since there are three thick brachial nerve bundles at the shoulder level of a forelimb, initially we severed all of them concomitantly with limb amputation as is routinely done in amphibians. In the denervated limbs, amputation calluses were formed as in innervated limb amputation (seven out of seven limbs; Fig. 5A). Although these nerves appeared to be the major brachial nerves, we performed another type of denervation to eliminate the possibility that there may still be a minor but effective number of nerves in the denervated limbs. In the second denervation experiment, the inside tissues of the limb, such as bones and muscles, were cut off at the elbow level after limb amputation and grafted underneath the skin near the shoulder. Even in such a severe condition, amputation calluses were still formed at the edge of the amputated bones (eight out of nine limbs; Fig. 5B, C), although the size of the calluses is largely impaired in most cases. These two types of denervation experiments suggest that the mechanism to initiate amputation callus formation is independent from the nerve signals in mice.

**Table 3:** Formation of the amputation and fracture calluses in denervated limbs

	Day after surgery	Number of samples	Normal	Partially formed^a^	Not formed^b^
Amputation callus					
Mouse					
Brachial plexus severed	7 days	7	7 (100%)	0 (0%)	0 (0%)
Limb tissue transplanted	7 days	9	1 (11%)	7 (78%)	1 (11%)
*Xenopus*					
Brachial plexus severed	10 days	6	6 (100%)	0 (0%)	0 (0%)
Fracture callus					
Mouse					
Brachial plexus severed	7 days	11	11 (100%)	0 (0%)	0 (0%)
*Xenopus*					
Brachial plexus severed	10 days	4	4 (100%)	0 (0%)	0 (0%)

^a^The size of calluses is dramatically reduced, but small cartilaginous or ossified tissues were found ectopically.

^b^No cartilaginous or ossified tissues were found ectopically.

In *Xenopus*, it is already known that surgically removing the three bundles of brachial nerves from the limb is enough to inhibit blastema formation (Korneluk et al. 1982; Endo et al. 2000). In that case, the amputation surface is covered by mature skin and a spike fails to form. When the stump was observed in denervated limbs, cartilaginous calluses were still observed to form as in the case of innervated limb amputation (six out of six limbs; Fig. 5D; see also Korneluk et al. 1982; Endo et al. 2000). Therefore amputation callus formation is nerve‐independent in *Xenopus*.

We also examined if callus formation in fracture healing is disturbed by limb denervation. When limbs were observed at 7 days in mice and 10 days in *Xenopus* after an incision was made in the forearm bones, fracture calluses were found in all cases (Table 3). It appeared that there was no obvious difference in morphology and size, suggesting that the early phase of callus formation, at least, proceeds independently of the nerve signals in both animals.

### The wound epithelium is less innervated in mice compared to *Xenopus*

Given the results in Figure 5 and Table 3, the callus‐forming response to limb amputation in mice and *Xenopus* appears to occur independently of signals from nerves. On the other hand, blastema formation is a nerve‐dependent event in amphibian regeneration, and it does not occur in mouse forearm amputation. Since nerves are known to be involved in the conversion of the WE to the functional AEC (Satoh et al. 2008; Simões et al. 2014), and the innervation of regenerating nerve endings is evident in the AEC (Suzuki et al. 2005; Satoh et al. 2008), we examined the distribution of regenerating axons from the surgically deviated nerves. In mice, however, the physical contact between the WE and nerves from the stump is prevented just after amputation. For example, a blood clot is formed on the stump and re‐epithelialization of the wound surface finishes late, compared to rapid wound closure in amphibians. In this study, to increase the probability of nerves making contact with the WE, we modified the accessary limb model (ALM) that was developed to investigate limb regeneration in the axolotl (for detailed protocol of an axolotl version, see Lee et al. 2013; Endo et al. 2015) and surgically deviated a nerve to come into contact with the epithelium covering the wound. We also performed the ALM in *Xenopus* as a control.

**Figure 6:**
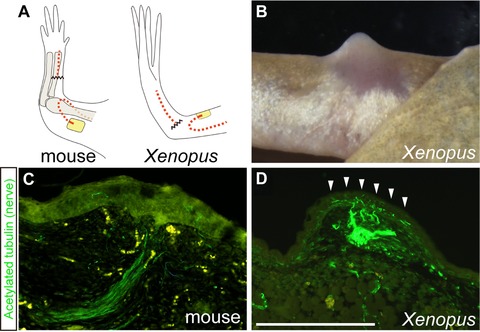
Distribution of deviated nerve fibers in the wound epithelium of mice and Xenopus. (A) Illustrations show the surgeries that each limb was given. By this surgery, blastema formation is induced in Xenopus (B) (2 weeks after surgery) but not in mice (not shown). (C), (D) Immunohistochemistry for nerve fibers in the WE. Compared to the intact epidermis of each animal, fewer nerve fibers were found in the WE of mice (C) (6 days after surgery), whereas the WE in Xenopus is more innervated (D) (7 days after surgery). The arrowheads indicate the WE innervated by the deviated nerves. Scale bar 500 μm (applicable to C).

As observed in the axolotl, when nerves were deviated into the full‐thickness skin wound of the *Xenopus* limb (see Fig. 6A), an ectopic blastema was induced (Fig. 6B; see also Mitogawa et al. 2014). In contrast, we did not observe formation of a blastema‐like structure in mouse limbs. We have tried more than 200 surgeries testing several surgical modifications but have not observed an ectopic outgrowth (not shown).

In *Xenopus*, the WE of nerve‐deviated skin wounds is more innervated than the normal skin in the adjacent non‐wounded area; however, this does not occur in wounds in mice. After a skin wound was made, the epidermal cells from the wound edge migrated and covered the wound surface within a day in *Xenopus* and after 3−4 days in mice. The distribution of nerve fibers around the WE was visualized by a nerve‐specific antibody. Since the connection between skin and other tissues is loose at the upper‐arm level in mice, in addition to the fact that mice move their limbs repeatedly, the surgically deviated nerves typically did not stay within the wound and were not observed underneath the WE in many cases. Nevertheless, we obtained 22 samples in which the deviated nerve stayed under the WE. We did not observe hyperinnervation of the WE in any of these limbs, compared to the normal epidermis. Even in the histological sections where deviated nerves were observed right next to the overlying WE, and appeared to elongate axons toward the epidermis, innervation from the deviated nerves was not found within it (Fig. 6C). On the other hand, in *Xenopus*, increased signals of a neural marker were seen in the WE just above the deviated nerves compared with normal skin (Fig. 6D), as is already shown in the WE after limb amputation (Suzuki et al. 2005).

## Discussion

### Amputation callus as a regenerative response—mouse versus *Xenopus*

In mice, a typical response to limb amputation in the middle of long bones is formation of the amputation callus. A similar cartilaginous tissue is seen also in amputated *Xenopus* limbs, which then grows distally to form the spike structure (Korneluk et al. 1982; Korneluk & Liversage 1984; Endo et al. 2000). We found that the amputation calluses in mice and *Xenopus* not only are morphologically similar but also show other similarities, such as cellular contribution to the growth, the response to Clo‐lipo injection, and denervation.

**Figure 7:**
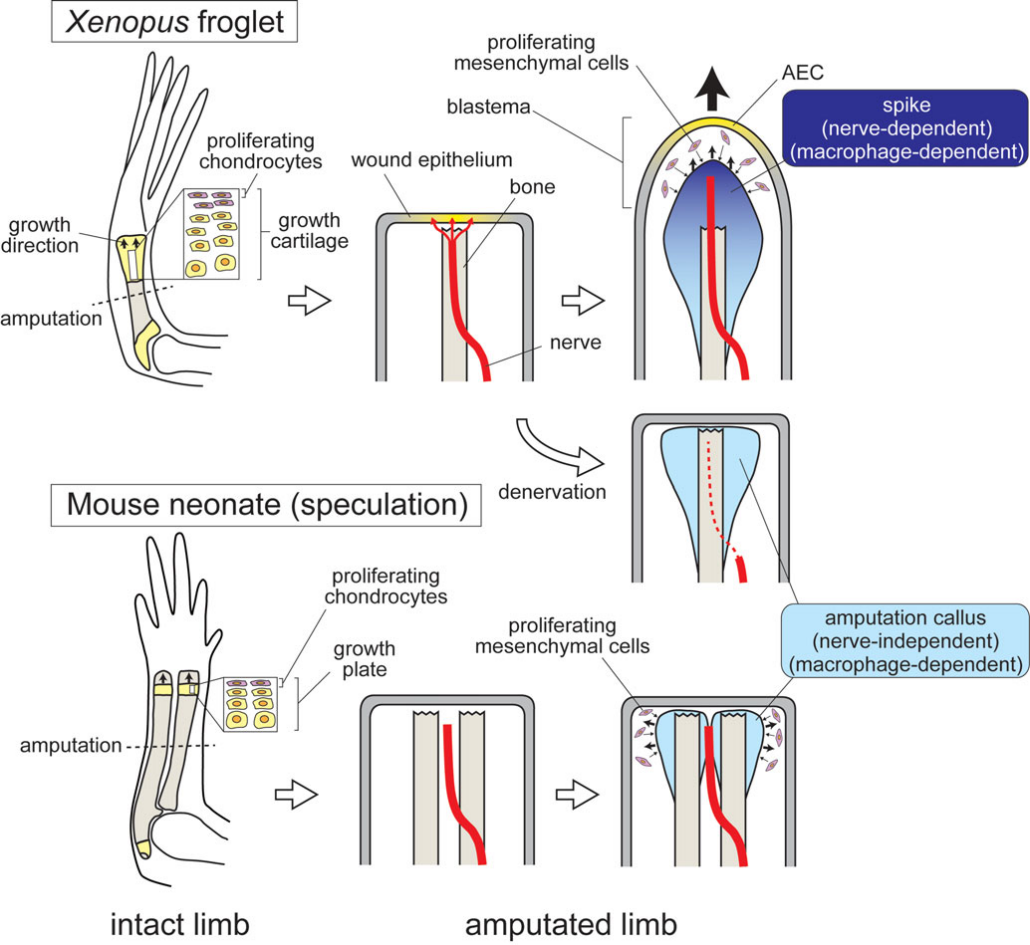
A schematic model of differences in nerve‐dependent and independent regenerative responses between Xenopus and mice. In Xenopus, regenerating axons innervate into the WE, and induce conversion of the WE into the AEC and conclusively formation of a blastema. In mice, however, axons do not innervate into the WE. Blastema formation does not occur, presumably because of failure in AEC formation (this is a speculation from the ALM experiment). Amputation callus formation at the bone terminus is a nerve‐independent and common response between mice and Xenopus.

In response to amputation, new cartilage forms from the periosteum and associated mesenchymal cells by proliferation and subsequent differentiation into chondrocytes that become incorporated into the regenerating cartilage (Figs 2 and 3). Bone growth in this manner is different from elongation of intact long bones. The outgrowth of long bones is mainly driven by proliferation of immature chondrocytes in the epiphyseal growth cartilages. Within the growth cartilages, chondrocytes divide and mature in a longitudinal direction in both mammals and frogs, resulting in bone elongation (Fig. 7) (Hunziker & Schenk 1989; Felisbino & Carvalho 1999; Miura et al. 2008). On the other hand, the growth of the amputation callus is driven by proliferation of undifferentiated mesenchymal cells, which eventually differentiate into chondrocytes in the callus. Several studies indicate that the growth plate regenerates at the amputated bone terminus in rat neonates (Libbin & Weinstein 1986, 1987), although we did not find regenerated growth plates in our mouse samples, perhaps because we focused only on the early phase. However, even if the growth plate could regenerate, formation of the amputation callus does not seem to be a result of regeneration of the growth plate because of the difference in cellular contribution between the amputation callus and growth plate.

Formation of the amputation callus is inhibited by Clo‐lipo injection during the early phase in mice and *Xenopus* (Fig. 4 and Table 1). Clo‐lipo injection is known to ablate macrophages (Van Rooijen 1989), and we confirmed that expression of macrophage markers was reduced after Clo‐lipo injection in mice. Given this correlation and the observation that macrophage depletion is associated with regeneration failure in several systems (Godwin et al. 2013; Petrie et al. 2014), we concluded that inhibition of the amputation callus probably resulted from reduction of the macrophage number, and that the inflammatory responses play crucial roles in forming the amputation callus in both animals. There are two possible functions of macrophages in callus formation. The first is that macrophages secret functional molecules to promote soft callus formation. Colony‐stimulating factor 1 is one of the candidate molecules (Raggatt et al. 2014). The second is that macrophages produce matrix metalloproteases (MMPs) (Webster et al. 2006) and that histolysis by MMPs affects the initiation of callus formation. MMPs degrade extracellular matrices (ECMs) and might free mesenchymal progenitor cells as a result. Proteolytic fragments of ECMs might also work as functional molecules to activate chondrogenesis or regenerative responses (Feng et al., 2005; Satoh et al., 2011). These events can also be a prerequisite for limb regeneration.

It is of interest whether or not limb denervation affects formation of the amputation callus, since denervation is one of the effective ways to inhibit limb regeneration when performed concomitantly with amputation. In *Xenopus*, when blastema formation is totally inhibited by denervation, the calluses are still formed as described previously (Fig. 5) (Korneluk et al. 1982; Endo et al. 2000). Amputation calluses in *Xenopus* denervated limbs have a similar morphology to the ones in mice, even when the mouse limb is not denervated. In addition, the ablation of nerves in two different ways did not completely inhibit amputation callus formation in mice (Fig. 5 and Table 2). These results suggest that nerves are not necessary to initiate amputation callus formation, unlike blastema formation which is nerve‐dependent.

### Amputation callus versus fracture callus versus spike

These characteristics are also applicable to the callus that is formed in fracture healing (a fracture callus). First, the fracture callus starts to be formed from the periosteum and is also derived from mesenchymal progenitor cells as well as the amputation callus (reviewed by Egawa et al. 2014). Second, a recent study shows that macrophage depletion using the genetically inducible mouse model inhibits fracture callus formation (Raggatt et al. 2014), which is consistent with the results of this study (Fig. 4 and Table 1). Third, although denervation of the limb tends to impair some fracture healing events such as mineralization, formation of the fracture callus occurs even in the absence of the nerve (Al‐Harby et al. 1996; Yegengil 2011; Song et al. 2012; Table 3). These similarities suggest that the fracture callus is formed by the same mechanism as the amputation callus.

A recent study using the ALM has demonstrated that a spike can be inducible from a skin wound on the lateral side of the limb by deviating nerves into the wound in *Xenopus* too (Mitogawa et al. 2014). In the *Xenopus* ALM study, unlike the axolotl ALM, a bone underneath the skin wound has to be injured to evoke the fracture healing responses necessary for the formation of the lateral spike. The fracture callus on the injured bone appears to grow toward the AEC and eventually forms a lateral spike (Mitogawa et al. 2014; see also Makanae et al. 2014b). Given the similarities between the fracture callus and amputation callus as described above, it is strongly suggested that the amputation callus is necessary for amputation‐induced spike formation.

### Nerve and wound epithelium—mouse versus *Xenopus*

The key mechanism that causes different regenerative responses to limb amputation between mice and *Xenopus* might be a different responsiveness to signals from nerves. Since previous studies show that innervation of the WE appears to be required for blastema formation (Suzuki et al. 2005; Satoh et al. 2008), the absence of nerve fibers in the mouse WE may be involved in failure of blastema formation (Fig. 7). The induction of the AEC from the WE is one of the possible functions of the nerves (Satoh et al. 2008; Stocum 2011; Kumar & Brockes 2012; Simões et al. 2014). An innervated WE is evident in regenerative and partially regenerative animals prior to induction of the blastema (Suzuki et al. 2005; Satoh et al. 2008). Some growth factors produced by the nerves, such as fibroblast growth factors and bone morphogenetic proteins, appear to induce formation of the functional AEC (Satoh et al. 2008; Makanae et al. 2014a). Once the AEC is formed, undifferentiated mesenchymal cells accumulate and proliferate to form a blastema (reviewed by Stocum 2011). As we discussed above, these blastema progenitor cells might arise from the stump tissues under the macrophage functions. The AEC directs the blastema growth distally. In mice, however, the amputation plane heals and eventually forms a scar instead of a blastema, as in *Xenopus* denervated limbs. A morphologically similar structure to the AEC is not found. In such non‐regenerating wounds, the basement membrane is reformed beneath the epidermis and is thought to interfere with the epithelial−mesenchymal interactions that are necessary to maintain blastema outgrowth (Neufeld & Day 1996; Endo et al. 2004; see also Seifert et al. 2012). Since the AEC is the signaling center for blastema outgrowth through epidermal−mesenchymal interaction, an AEC defect would result in failure of blastema formation (see Tassava & Olsen 1982).

It is not possible to make a simple comparison between mammals and amphibians because there are so many differences in physiology and genetics. The ALM experiment in this study, however, suggests the possibility that the WE and nerves cannot interact with each other in mice, even if they are experimentally placed side by side. In certain conditions, mammals can regenerate injured axons of peripheral nerves (reviewed by Snider et al. 2002). When skin is wounded, peripheral nerves sprout into the damaged area, which is especially prominent in neonates (De Lima et al. 1999; Beggs et al. 2012). Therefore it does not appear that there is a difference in axon regenerative potency between mammals and amphibians. The mechanism that does not allow deviated nerve fibers to innervate into the WE in mice is still to be determined.

## Materials and Methods

### Ethics statement

All animal experiments were performed in accordance with protocols approved by the Institutional Animal Care and Use Committee of Aichi Gakuin University, Division of Liberal Arts and Sciences.

### Animals

ddY mice and *Xenopus laevis* froglets were purchased from Chubu Kagaku Shizai Co. Ltd (Nagoya, Japan). Parental mice were bred and maintained under standard conditions in the laboratory, and neonatal mice were used in experiments. *Xenopus* were maintained at 20−22°C.

### Surgical procedures

Postnatal day 1 (P1) mice were anesthetized by placing on ice for 5−10 min for the following surgical procedures. For all mouse experiments, only one side forelimb was used in each neonate. After surgery, neonates were placed at room temperature until they stopped bleeding and became warmed to normal body temperature, and were then put back to the mother.

In the amputation model, limbs were amputated with a surgical knife at the middle of the forearm level, and protruding bones were trimmed with ophthalmic scissors. In the skeletal defect model, skin was incised in the middle of the forearm, and a single incision was made in the radius and ulna with ophthalmic scissors avoiding muscle damage.

To investigate nerve dependence for amputation callus formation, we performed two types of denervation experiments. In the first experiment, the brachial plexus was cut at the ventral side of the shoulder level before limb amputation. In the second denervation experiment, a limb was amputated at the middle of the forearm level, and the connection between the skin and muscles of the forearm was carefully severed. Surrounding skin was pulled down to expose the internal tissues, such as bones and muscles, which were excised and immediately grafted underneath the skin at a more proximal level near the shoulder (see the illustration in Fig. 5B), thus ensuring that the amputated and grafted skeletal tissues were not innervated.

In the nerve deviation experiment, a piece of skin was removed from the middle of the forearm level by a biopsy punch (1.5 mm in diameter). The median nerve was carefully pulled out from the cubital fossa and rerouted beneath the skin to a prepared full‐thickness skin wound. Terramycin ointment (Yoshindo, Toyama, Japan) and Tegaderm Film (3M Health Care, Neuss, Germany) were used to care the wounds deviated nerves.

*Xenopus* froglets were anesthetized in 0.1% MS222 (Sigma St. Louis, MO. USA) solution for surgical procedures. The amputation model and fracture model were carried out in the same manner as in mice. In the nerve deviation experiment, three thick bundles of brachial nerves were cut at the elbow level and rerouted beneath the skin to a prepared full‐thickness skin wound. Froglets were placed in a wet condition overnight at 4°C after the nerve deviation surgery. In the denervated limb amputation experiment, brachial nerves were cut at the wrist level and withdrawn proximally at the upper arm region. Then limbs were amputated with a surgical knife at the middle of the forearm level. Post‐surgery, *Xenopus* were maintained in 12.5% Holtfreter's solution.

### Macrophage depletion

In mice, for macrophage depletion during the early phase of the amputation responding events, 10 μL of liposome‐encapsulated clodronate (Clophosome [neutral] [7 mg/mL clodronate]; FormuMax Scientific, USA) was injected into the forelimb subcutaneously at P0, P1, and P2 prior to amputation. Then the limbs were amputated at the middle of the forearm 9−12 h after injection at P2. In *Xenopus*, either limb subcutaneous injection or intraperitoneal injection was made 3, 2, and 1 day(s) before amputation. For intraperitoneal injection, 10 μL/g body weight of Clophosome was prepared. For controls, plain liposomes (Plain control liposome for Clophosome [neutral], FormuMax Scientific, USA) were used.

### Whole‐mount skeletal staining

The whole‐mount skeletal staining in Figure 1 was performed with alcian blue 8GX and alizarin red S (Wako, Osaka, Japan) according to Ide (2012). Briefly, samples were fixed in 100% ethanol and stained with 70% ethanol containing 5% acetic acid, 0.015% alcian blue and 0.005% alizarin red at 37°C. Samples were then treated with 1% KOH and cleared with glycerol.

### Histology

For the histological sections in Figures 2−5, mouse samples were fixed in 4% paraformaldehyde phosphate‐buffered saline (PBS) overnight at 4°C, and *Xenopus* samples were fixed in 10% formalin PBS overnight at room temperature (RT). Fixed samples were washed in PBS and decalcified in Morse's solution (10% sodium citrate and 22.5% formic acid) overnight at RT, processed, embedded in paraffin, and sectioned (10 μm). Tissue sections were processed, treated with 3% acetic acid, and stained with 1% alcian blue 8GX (Sigma) for 1 min. After alcian blue staining, slides were washed in 3% acetic acid and PBS for a few minutes, and processed for hematoxylin and eosin staining.

### Immunohistochemistry

For acetylated‐tubulin immunohistochemistry, tissue sections were de‐paraffinized and rehydrated in Tris‐buffered saline (TBS). After treated with Blocking One Histo (Nacalai tesque, Kyoto, Japan) for 10 min at RT, sections were incubated with mouse anti‐acetylated tubulin monoclonal antibody (Sigma #T7451, diluted 1:50) for 1 h at 37°C. For mouse samples, the primary antibody was directly labeled with Alexa 488 (Invitrogen). For *Xenopus* samples, sections were incubated with Alexa 488 labeled secondary antibody (Invitrogen, diluted 1:400).

For type II collagen immunohistochemistry, mouse tissue sections were treated with 0.001% proteinase K (Invitrogen) in TBS for 10 min at 37°C, and incubated with rabbit anti‐collagen II polyclonal antibody (Abcam #ab53047, diluted 1:100) for 1 h at 37°C. *Xenopus* tissue sections were treated with 0.0005% proteinase K in TBS for 25 min at RT, and incubated with mouse anti‐collagen II monoclonal antibody (Developmental Studies Hybridoma Bank, #II‐II6B3, diluted 1:100) for 1 h at 37°C. After primary antibody incubation, both sections were incubated with Alexa 488 labeled secondary antibody.

### BrdU incorporation

Animals received intraperitoneal injections of BrdU (Roche, 50 μg/g of body weight) and were sacrificed at particular timings. Samples were processed for tissue sections. For detection, sections were treated with 4 mol/L HCl at RT for 15 min and 0.001% proteinase K in TBS for 10 min at 37°C. Then the sections were incubated with rat anti‐BrdU antibody (Abcam, diluted 1:400) for 60 min at 37°C. After washing in TBS, tissue sections were incubated with Alexa 555 or Alexa 488 labeled secondary antibody for 45 min at RT.

### Statistical analysis

Statistically significant differences were determined using Fisher's exact test. *P* values were calculated using the software R version 3.1.3, and *P* < 0.05 was considered statistically significant.
